# Carbazole–benzocarbazole fragments having derivative as very efficient host material for TADF based OLEDs

**DOI:** 10.1039/d5na01189b

**Published:** 2026-02-16

**Authors:** Sushanta Lenka, Daiva Tavgeniene, Hsuan-Min Wang, Jayachandran Jayakumar, Dovydas Blazevicius, Gintare Krucaite, Yu-Lin Chi, Saulius Grigalevicius, Jwo-Huei Jou

**Affiliations:** a Department of Materials Science and Engineering, National Tsing Hua University No. 101, Section 2, Guangfu Rd, East District Hsinchu 30013 Taiwan jjou@mx.nthu.edu.tw; b Department of Polymer Chemistry and Technology, Kaunas University of Technology Radvilenu Plentas 19 LT50254 Kaunas Lithuania saulius.grigalevicius@ktu.lt; c Department of Chemistry, National Tsing Hua University No. 101, Section 2, Guangfu Rd Hsinchu 30013 Taiwan

## Abstract

Organic light-emitting diodes (OLEDs) are widely adopted in modern display and lighting technologies; however, while significant progress has been achieved in the development of thermally activated delayed fluorescence (TADF) emitters, comparatively limited attention has been paid to the rational design of compatible host materials. In this work, a carbazole–benzocarbazole-based derivative, BCCOX, was designed and synthesized as an efficient host material for green TADF OLEDs. BCCOX maintains excellent thermal integrity, exhibiting a decomposition onset beyond 400 °C and a high glass transition temperature of approximately 194 °C, ensuring morphological integrity and operational reliability under device fabrication and operating conditions. The material further possesses a wide optical bandgap of 3.6 eV and favorable exciton dynamics, enabling effective exciton confinement and utilization in TADF systems. When employed as the host matrix in green TADF OLED devices, BCCOX delivers a maximum external quantum efficiency of 10.4%, along with a current efficiency of 20.6 cd A^−1^ and a power efficiency of 12.7 lm W^−1^ at relatively low driving voltages. These results demonstrate that carbazole–benzocarbazole architectures offer a robust and versatile platform for high-performance host materials, positioning BCCOX as a promising candidate for efficient, stable, and cost-effective next-generation OLED display applications.

## Abstract 2

OLEDs are widely used in displays and lighting, yet host material design for TADF systems remains limited. Here, a carbazole–benzocarbazole derivative, BCCOX, is developed as an efficient host for green TADF OLEDs.

## Graphical abstract

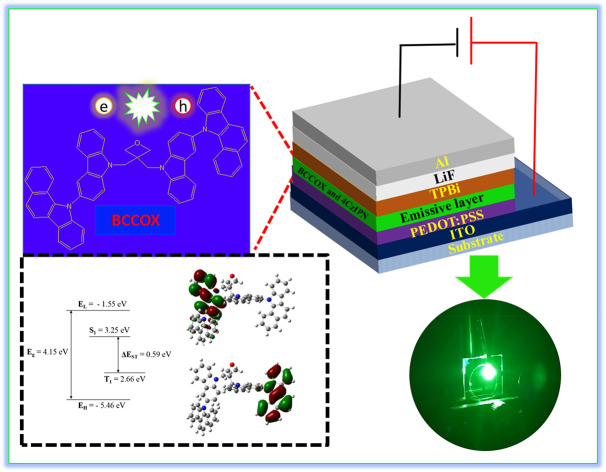

## 1. Introduction

Over the past several decades, OLEDs have attracted significant interest owing to their unique benefits, positioning them as a core technology for advanced display systems and solid-state lighting.^1–3^ Features such as broad viewing angles, superior contrast performance, and outstanding mechanical compliance have enabled the successful commercialization of unconventional display architectures, including flexible, bendable, and contoured forms.^4–6^ In addition, OLEDs function effectively under low operating voltages, which contributes to lower energy consumption, extended operational lifetimes, and strong compatibility with portable and wearable electronic applications.^7–9^ Their cost-effective fabrication by both vacuum and solution processes also supports large-area scalability.^10,11^ Realizing their full commercial potential, however, requires advances in device engineering, modeling, and especially novel organic material design. Based on the nature of the emitting species, OLED architectures are generally categorized into fluorescent, phosphorescent, and TADF systems.^12–14^ In recent years, TADF-based and hyperfluorescent OLEDs have attracted considerable research interest, as they offer a compelling combination of high internal quantum efficiency, superior color purity, and reduced material cost. Following the seminal contributions of Adachi and co-workers, a wide variety of TADF emitters have been reported.^15–17^ Nevertheless, the development of compatible host materials tailored specifically for TADF applications has lagged behind, and the selection of suitable hosts remains comparatively narrow.

At present, the majority of TADF OLEDs continue to employ traditional host materials—such as CBP, mCP, mCBP, PPT, and DPEPO—that were originally optimized for phosphorescent devices.^18^ Host materials for TADF emitters can be broadly divided into bipolar single-component systems and composite hosts formed by blending hole-transporting and electron-transporting materials.^19^ Bipolar hosts are particularly effective in minimizing charge trapping by reducing interfacial discontinuities, whereas mixed unipolar systems can improve charge injection and carrier balance within the emitting layer. Among the various host platforms explored, carbazole-based materials have emerged as one of the most extensively investigated families. These include carbazole-derived unipolar hosts, carbazole–dibenzothiophene bipolar systems, carbazole–polyaromatic hybrids, carbazole–silane-based architectures, carbazole hosts incorporating cyano-substituted acceptors, carbazole–phosphine oxide hybrids, and cyanocarbazole derivatives.^20–22^ In addition to carbazole-centered systems, several alternative host material classes have been examined, including phosphine oxide-based compounds, triphenylamine-derived bipolar hosts, heterocyclic frameworks, and even self-emissive TADF materials capable of functioning as both emitter and host.^23^ Despite these advances, only a limited subset of conventional and exciplex-type hosts has demonstrated consistently high compatibility with TADF emitters. Notably, the number of efficient host materials specifically designed for solution-processed TADF OLEDs remains particularly limited, underscoring the necessity for continued molecular innovation in this area. For an OLED to achieve high efficiency and operational stability, the host material must satisfy several stringent requirements. Foremost among these is a sufficiently high triplet energy level relative to that of the emitter, which is essential to suppress reverse energy transfer and maintain exciton confinement. In addition, an effective host should possess robust thermal stability, good morphological integrity in thin films, and appropriately positioned highest occupied molecular orbit (HOMO) and lowest unoccupied molecular orbit (LUMO) energy levels to enable efficient charge injection, balanced carrier transport, and low driving voltage. Bipolar host compounds, containing both electron-rich and electron-deficient units within the same molecule, provide distinct benefits compared to unipolar hosts by enabling simultaneous transport of holes and electrons in the emissive layer, which improves charge balance and extends device operational stability.^24^ In donor–acceptor molecular architectures, hole-transporting segments like triphenylamine, phenoxazine, and notably carbazole are commonly employed.^25–28^ Carbazole is particularly attractive due to its rigid molecular backbone, excellent chemical and thermal robustness, and high triplet energy, making it a highly effective electron-donating building block for the construction of high-performance bipolar host materials.

We describe the rational design and successful synthesis of a new host material based on a carbazole–benzocarbazole framework, 3,3-bis[3-(benzo[*a*]carbazol-11-yl)carbazol-9-ylmethyl]oxetane (BCCOX), featuring a rigid oxetane-linked architecture that integrates high thermal robustness with favorable electronic properties, achieved with an excellent yield of 64%. BCCOX was employed as the host matrix in OLEDs using the green emitter (4CzIPN from 1 to 10 wt%), enabling a systematic study of host–guest interactions, energy transfer efficiency, and charge balance. The device containing 10 wt% 4CzIPN delivered the best overall performance, reaching a maximum EQE of 10.4% and a peak luminance above 7500 cd m^−2^, which reflects efficient exciton utilization and well-balanced charge transport within the emissive layer. These results position BCCOX as a promising high-efficiency, fabrication-friendly host for advanced TADF OLEDs.

## 2. Result and discussion

### 2.1. Synthesis protocol of BCCOX material

**Scheme 1:**
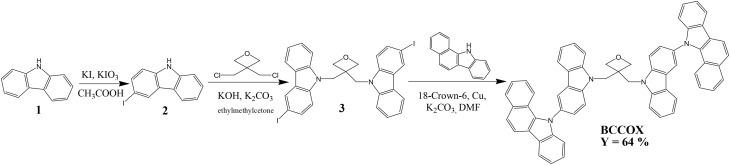
Synthesis of BCCOX.

The objective material: 3,3-bis[3-(benzo[*a*]carbazol-11-yl)carbazol-9-ylmethyl]oxetane (BCCOX) was synthesized during the synthesis steps as shown in Scheme 1. 3-Iodo-9*H*-carbazole (2) was firstly obtained from 9*H*-carbazole (1) by using the iodination method of Tucker. 3,3-Di[3-iodocarbazol-9-ylmethyl]oxetane (3) was then prepared by reaction of 3,3-di(chloromethyl)oxetane with an excess of the iodo-derivative 2. The objective compound BCCOX was obtained by Ullmann reaction of the diiodo-compound 3 with an excess of 11*H*-benzo[*a*]carbazole. All the prepared derivatives were confirmed by MS spectrometry, ^1^H and ^13^C NMR spectroscopy. The synthesized objective material is well soluble in conventional organic solvents.

### 2.2. Surface morphological characteristics

Fig. 2a–d illustrates AFM images of thin films composed of BCCOX-based host materials blended with varying weight percentages of the commercial emitter 4CzIPN, deposited *via* spin-coating onto ITO substrates. This study provides critical understanding into surface topography and roughness parameters (Fig. 2e–h), both of which play a pivotal role in determining the performance of OLED devices. Fig. 2a illustrates the surface topography of a film doped with 4CzIPN (1 wt%), showing moderate surface irregularities with an *R*_a_ of 0.96 nm and *R*_q_ of 1.21 nm. Increasing the emitter concentration leads to a gradual decrease in surface roughness, reflecting enhanced film uniformity. For example, the 3 wt% 4CzIPN film (Fig. 2b) exhibits slightly lower *R*_a_ and *R*_q_ values of 0.85 nm and 1.13 nm, respectively, while the 10 wt% 4CzIPN film (Fig. 2d) presents the smoothest surface in the series, with *R*_a_ and *R*_q_ measured at 0.78 nm and 1.02 nm, respectively. At 5 wt% (Fig. 2c), the film retains a relatively smooth morphology with *R*_a_ = 0.80 nm and *R*_q_ = 1.04 nm. This observation indicates that higher emitter concentrations improve the uniformity of the emissive layer's morphology, potentially by promoting improved molecular packing and mitigating interfacial roughness inherited from the underlying ITO layer. A smoother film morphology contributes to several performance benefits in OLEDs, including reduced surface defects and phase separation, enhanced interfacial contact between layers, and improved charge injection and balance.^29^ Furthermore, the more uniform emissive matrix supports effective energy transfer and exciton confinement, reducing non-radiative losses. Reduced surface roughness minimizes interfacial optical scattering, which in turn enhances light extraction and boosts overall device performance.^30,31^

### 2.3. Photophysical characteristics

UV-vis measurement was employed to examine the optoelectronic properties of the synthesized host compound BCCOX. As shown in Fig. 3a and b, the absorption and transmission profiles demonstrate pronounced optical behavior, featuring a well-defined absorption maximum at 295 nm, along with excellent optical transparency in the visible range, where the transmittance approaches ∼95% (Fig. 3b). These spectral features indicate that BCCOX exhibits dual optical behavior—effective absorption of UV radiation coupled with excellent transparency in the visible range—which is advantageous for OLED applications, where high transparency and efficient light emission are critical parameters.^32^ The bandgap of BCCOX was estimated to be nearly 3.66 eV, as derived from the Tauc plot method (Fig. 3c), wherein the extrapolation of the linear region of the (*αhν*)^2^*versus* photon energy (*hν*) plot intersects the energy axis. This wide optical bandgap further supports its suitability as a host material for blue or high-energy emitters in OLEDs. The superior transparency of BCCOX in the visible region not only facilitates enhanced light outcoupling but also aligns well with the requirements of solution-processed OLED architectures, contributing to improved device efficiency.^33^ Moreover, the ability of BCCOX to absorb UV light serves a protective function by shielding the underlying active layers from potential photodegradation, thereby contributing to the prolonged operational reliability of the device. Overall, these findings highlight the potential of BCCOX as a promising host material for the EML in OLED devices, offering a synergistic combination of optical transparency, UV-filtering capability, and favorable energy alignment conducive to high electroluminescence performance.

Ultraviolet Photoelectron Spectroscopy (UPS) was utilized to investigate the electronic structure of the BCCOX:4CzIPN composite system, providing essential insights into energy level alignment and interfacial electronic properties. UPS, owing to its high surface sensitivity, allows precise evaluation of key electronic parameters, including the valence band maximum (VBM), work function, and surface electronic states, which are crucial for optimizing materials used in electronic and optoelectronic devices.^34,35^ For composite thin films, UPS is particularly effective in examining interfacial electronic interactions, determining energy-level alignment with neighboring functional layers, and clarifying how compositional changes or molecular doping affect the electronic structure. In the present work, the HOMO level of the BCCOX:4CzIPN composite film was determined from the UPS spectra by linearly extrapolating the secondary electron cutoff and the leading edge of the photoemission onset (Fig. S4d). The HOMO level was determined to be 5.26 eV. LUMO energy level was subsequently estimated using the relation *E*_LUMO_ = −*E*_HOMO_ + *E*_g_, where *E*_g_ denotes the optical bandgap derived from the Tauc plot (Fig. S4b). The calculated optical bandgap was 3.17 eV, leading to a corresponding LUMO energy level of 2.09 eV. The integrated evaluation of UPS and UV-vis spectroscopic results confirms that the HOMO, LUMO, and work function of the 4CzIPN:BCCOX system are suitably matched with the energy levels of the neighboring charge-transport layers, reflecting favorable interfacial alignment and compatibility. This favorable alignment facilitates efficient charge injection and transport across interfaces, a critical requirement for high-performance organic optoelectronic devices. These findings underscore the potential of BCCOX-based composites as host materials in the emissive layer (EML) of OLEDs, where precise energy level matching is essential for optimizing electroluminescent efficiency and device stability.

To comprehensively evaluate the photophysical behavior of the novel host material BCCOX, TRPL and LTPL analyses were carried out. These results (Fig. 3d–f) provide detailed information on exciton kinetics by resolving prompt and delayed emission components, which is essential for assessing TADF characteristics. Analysis of the decay dynamics reveals the balance between radiative and non-radiative recombination pathways, the efficiency of host-to-guest energy transfer, and the possible involvement of trap states or interfacial imperfections, all of which critically influence overall OLED device performance.^36^ Furthermore, the quality and uniformity of the emissive thin film can be inferred from the decay characteristics, reflecting the material's morphological integrity and purity. Complementary LTPL measurements were performed under cryogenic conditions to suppress thermal quenching and reveal the intrinsic emission characteristics of BCCOX. This approach facilitates the sensitive identification and analysis of triplet-state emissions, defect-associated emissive states, and charge-transfer-related luminescence. At the same time, it offers a more comprehensive understanding of underlying molecular aggregation behavior and phase-separation processes, which are often thermally suppressed or indistinguishable under conventional room-temperature measurement conditions. Notably, *E*_T_ of BCCOX was determined to be 2.80 eV, indicating its compatibility with a range of high-energy phosphorescent or TADF emitters and confirming its potential as an effective host material in multilayer OLED architectures. The transient photoluminescence decay measurements were conducted under deoxygenated conditions by purging the sample chamber with nitrogen before measurement, thereby substantially reducing oxygen-induced quenching. Overall, analyses from TRPL and LTPL reveal detailed information on exciton recombination dynamics, emission behavior, and the energy landscape of the emissive layer. These findings highlight the capability of BCCOX to support efficient exciton confinement, balanced charge transport, and reduced non-radiative losses—key attributes that substantiate its promise for integration into next-generation OLED devices with enhanced operational efficiency and stability.

### 2.4. Thermal properties

Differential scanning calorimetry (DSC) and thermogravimetric analysis (TGA) were used in order to investigate behavior under heating of the objective host derivative BCCOX. It was confirmed that the material has characteristic of high thermal stability. As it is obvious from the Fig. 4a, temperature of thermal destruction for the derivative exceeded 400 °C as confirmed by the TGA method. It is known that the oxetanyl group containing molecules can form hydrogen bonding in the solid state, and the material demonstrated high thermal stability as well as high glass transition temperature due to the intermolecular interaction.^37^ On the other hand, the oxetanyl groups start thermal polymerization during the TGA experiment at about 275 °C, which is confirmed by DSC measurement. Oligomeric products with higher thermal stability are formed during the polymerization process. It should be also stated that the compound has a residue of moisture and solvents, which was registered in the TGA investigation.

The DSC analysis is shown in Fig. 4b. Following synthesis and purification, BCCOX was isolated as an amorphous solid. In the initial DSC heating cycle, the material transitions to a liquid state at a glass transition temperature (*T*_g_) of approximately 194 °C, after which polymerization begins around 275 °C, leading to the formation of oligomeric structures. This is well confirmed during the second DSC heating scan. It is evident that the formed mixture of the oligomers has only a glass transition of about 200 °C.

### 2.5. Electrochemical studies and DFT calculation

Electrochemical characterization of the synthesized BCCOX host material was performed in acetonitrile (MeCN) at a concentration of 1 mM to examine its redox properties. All measurements were carried out under standard conditions using a conventional three-electrode setup, consisting of a glassy carbon (GC) disk electrode with a diameter of 3.0 mm as the working electrode, a platinum wire and a non-aqueous Ag/AgNO_3_ (0.01 M) electrode were used as the counter and reference electrodes, respectively. Tetrabutylammonium hexafluorophosphate ([NBu_4_][PF_6_], 0.1 M in MeCN) was used as the supporting electrolyte to ensure adequate ionic conductivity. All measurements were performed at room temperature with a scan rate of 100 mV s^−1^. The electrochemical data thus obtained were utilized to estimate the HOMO energy levels of the BCCOX host material. The frontier orbital energy levels were estimated from cyclic voltammetry (CV) measurements. The HOMO level was calculated using the relation *E*_HOMO_ = −(*E*^ox^_1/2_ + 4.8) eV,^38^ where *E*^ox^_1/2_ corresponds to the onset oxidation potential derived from the CV curve. The LUMO level was then determined from the optical band gap according to *E*_LUMO_ = *E*_HOMO_ + *E*^optical^_g_. Based on the experimentally obtained oxidation onset at 0.72 V (Fig. 5b), the HOMO energy was determined to be −5.52 eV. Using the measured optical bandgap of 3.66 eV, the corresponding LUMO energy was subsequently derived as −1.86 eV. To gain deeper insight into the correlation between the electrochemical behavior and the molecular structure, DFT calculations were carried out. These calculations provided additional insight into the electronic structure and charge distribution characteristics of the compound, offering a comprehensive understanding of its potential suitability for optoelectronic applications.

All computations were performed using the Gaussian09 software package, employing the B3LYP exchange–correlation functional in conjunction with the 6-31G basis set. Solvent effects were incorporated using the polarizable continuum model (PCM), with toluene specified as the solvent medium, consistent with the default configuration in Gaussian09. Frontier molecular orbital analysis indicates that the HOMO is mainly concentrated on the benzocarbazole moiety, whereas the LUMO is largely situated on the carbazole unit. This clear spatial separation between the HOMO and LUMO points to a strong intramolecular charge-transfer (CT) character. Supporting this, natural transition orbital (NTO) analysis shows that the electron density of the HOMO and LUMO resides on different carbazole-derived substituents. The computed energy levels of the HOMO and LUMO (see Table 1) were −5.46 eV and −1.55 eV, respectively, yielding an optical bandgap (*E*_g_) of 3.91 eV. Furthermore, the first singlet (S_1_) and triplet (T_1_) excited-state energies were estimated to be 3.25 eV and 2.66 eV, respectively, resulting in a singlet–triplet energy gap (Δ*E*_ST_) of 0.59 eV. These theoretical insights provide a valuable understanding of the compound's potential as a functional material in optoelectronic applications.

**Table 1:** Comparative study of HOMO and LUMO level obtained from electrochemical analysis and DFT study

Cyclic voltammetry			DFT study		
*E*_HOMO_ (eV)	*E*_LUMO_ (eV)	*E* ^optical^_g_ (eV)	*E*_HOMO_ (eV)	*E*_LUMO_ (eV)	*E* ^optical^_g_ (eV)
−5.52	−1.86	3.66	−5.46	−1.55	4.15

## 3. Electroluminescence studies

**Fig. 1:**
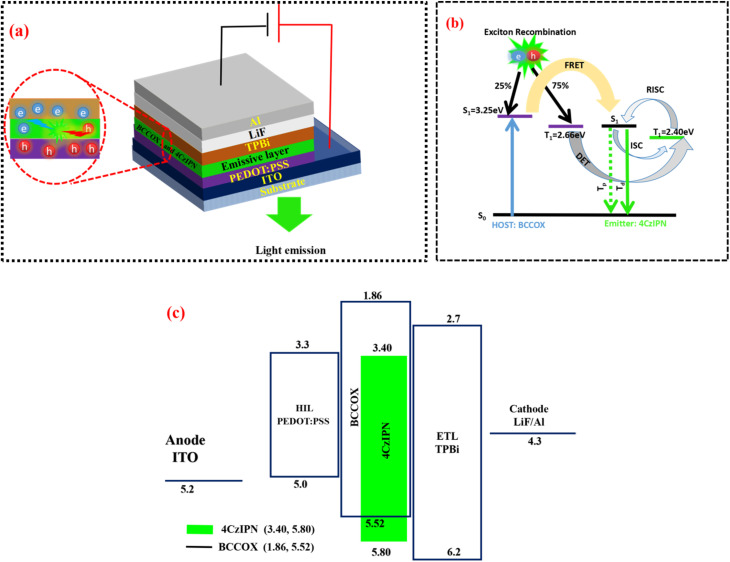
(a) Schematic illustration of an OLED device employing 4CzIPN as the emissive dopant within a BCCOX host framework; (b) representation of host-to-dopant energy-transfer pathways in the BCCOX system; and (c) schematic energy-level alignment of the individual functional layers comprising the OLED architecture.

In the final stage of the study, the electroluminescent properties of the devices were systematically investigated to evaluate their performance. Fig. 1c presents a detailed schematic of the complete OLED device architecture, offering a thorough depiction of the energy level alignment across all constituent layers. Particular emphasis was placed on the energetic correspondence between the HOMO and LUMO levels of the green TADF emitter and the host material, BCCOX. This careful alignment is important for ensuring efficient charge injection, exciton confinement, and overall device performance, as it directly influences the efficiency of energy transfer and the suppression of unwanted recombination pathways within the emissive layer. The analysis shows that the emitter's frontier orbitals are well contained within the energy range of the host, indicating excellent energetic compatibility. This strategic host–emitter pairing is intended to prevent reverse transfer of triplet excitons from the emitter to the host, a process effectively suppressed by the high triplet energy of BCCOX (2.80 eV). Such a high triplet energy is pivotal for maintaining exciton confinement within the emitter, thereby enhancing device stability and electroluminescence efficiency.^39^ Further experimental results, provided in the SI (Table S1 and Fig. S5), underscore the detrimental effects observed in control devices lacking guest doping or utilizing pristine emissive layers. These devices exhibit marked performance degradation, primarily attributed to imbalanced charge mobility and severe aggregation-induced quenching—phenomena commonly associated with undoped TADF systems. Collectively, these findings underscore the critical role of host–guest energy level alignment in achieving efficient exciton confinement and highlight the substantial impact of appropriate host selection on overall OLED performance and operational longevity.

**Fig. 2:**
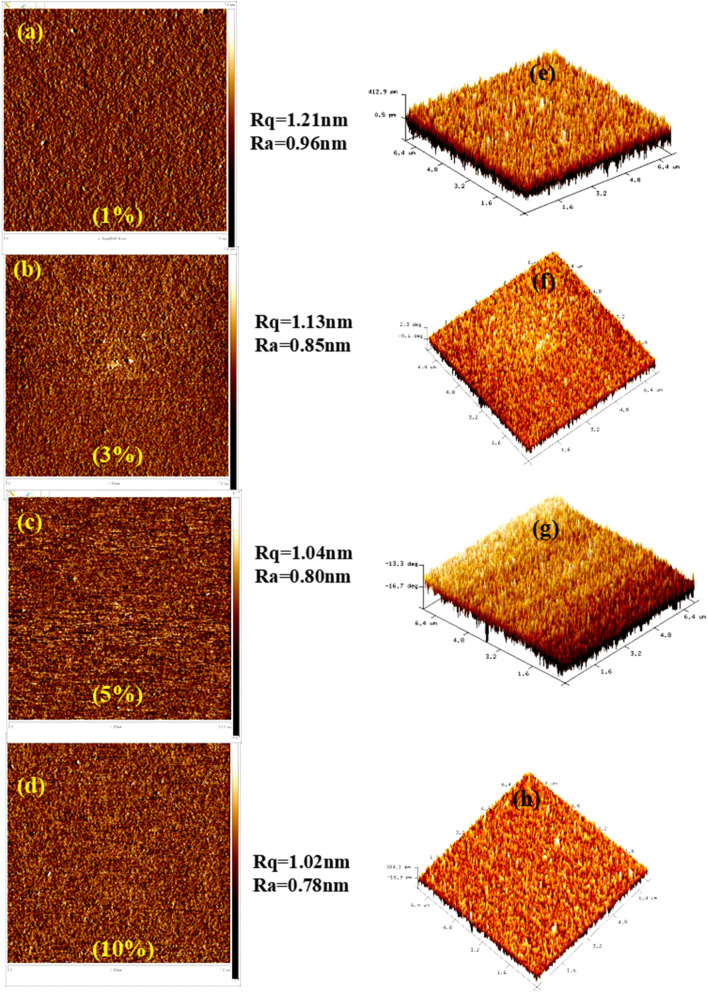
(a–d) AFM images of BCCOX host films doped with different concentrations of 4CzIPN: (a) 1%, (b) 3%, (c) 5%, and (d) 10%, illustrating the surface morphology and average roughness of each sample. (e–h) The corresponding three-dimensional AFM topographical representations of the films.

**Fig. 3:**
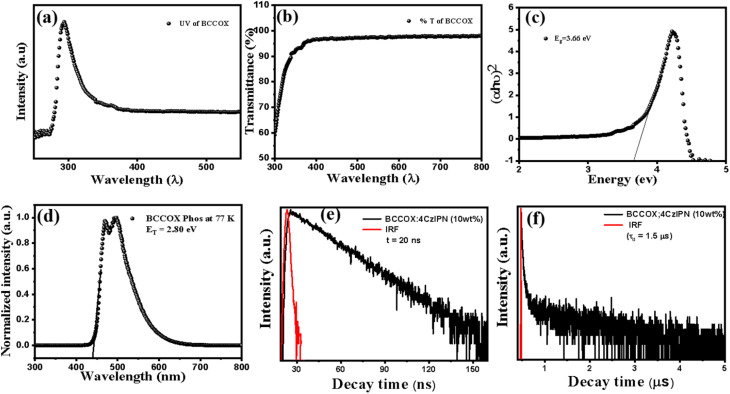
(a–c) UV analysis of BCCOX, (d) LTPL of BCCOX. (e and f) TRPL measurement of prompt and delayed of BCCOX under ambient conditions, respectively.

**Fig. 4:**
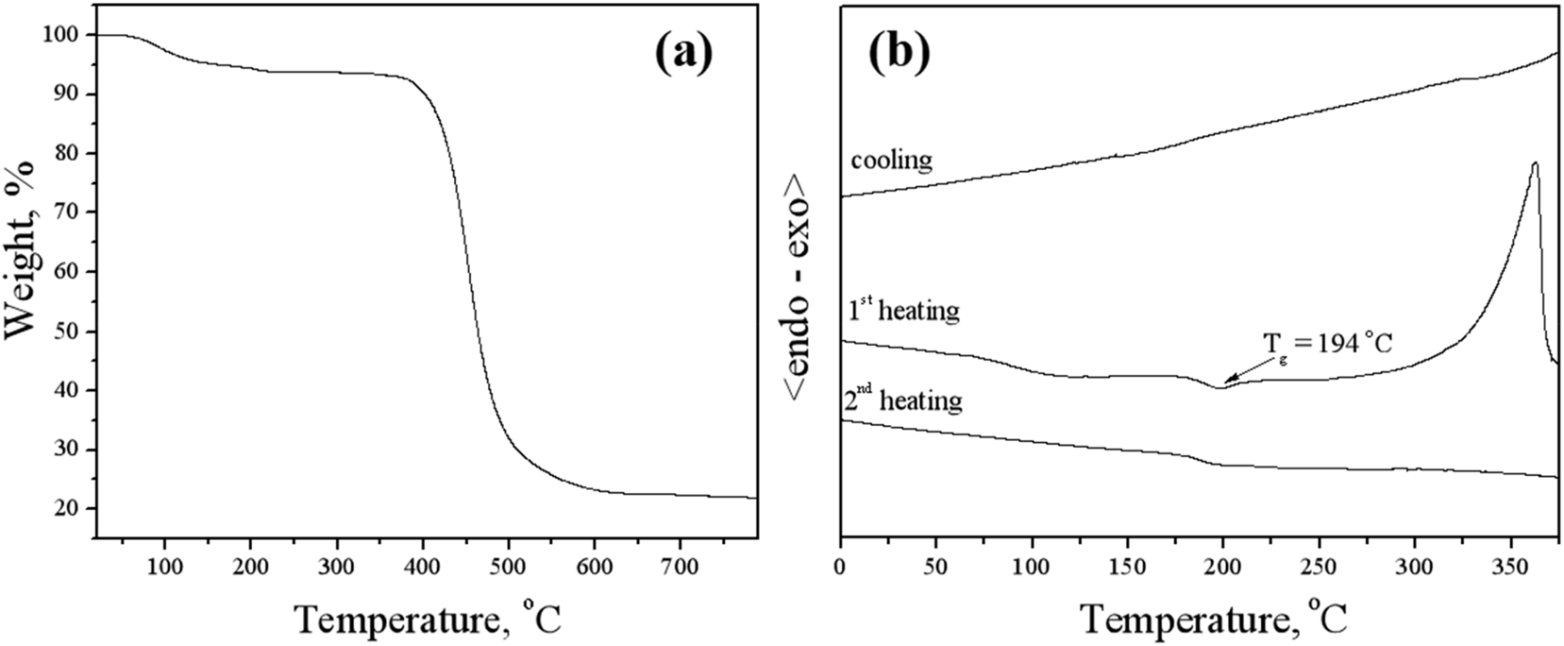
(a) Thermogravimetric analysis (TGA) of the BCCOX compound recorded at a heating rate of 10 °C min^−1^. (b) Differential scanning calorimetry (DSC) curves of BCCOX, measured with a heating rate of 10 °C min^−1^.

**Fig. 5:**
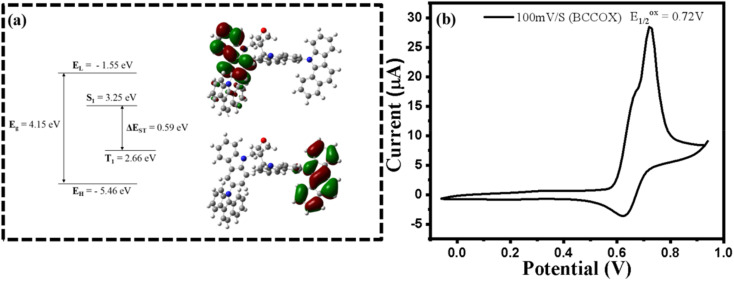
(a) Frontier molecular orbitals of BCCOX, illustrating the HOMO and LUMO energy levels, denoted as *E*_H_ and *E*_L_, respectively, accompanied by the associated energy band schematic; (b) cyclic voltammetry profile of BCCOX.

The issue of inefficient energy transfer in OLED architectures was effectively mitigated by employing the newly developed host material, resulting in enhanced operational characteristics of doped devices. As illustrated in Fig. 7a and S5, the EL spectra of devices with and without dopants display distinct spectral variations under different operating voltages. When the dopant level is low, energy transfer from the host to the guest emitter is incomplete, leading to increased non-radiative decay pathways and reduced device efficiency.^40^ By contrast, higher doping concentrations significantly promote host-to-guest energy transfer, as reflected by the pronounced green emission peak associated with the 4CzIPN emitter, signifying more efficient exciton utilization within the host materials BCCOX. These findings underscore the significance of optimizing dopant concentration to tailor device characteristics. An appropriate dopant level is crucial not only for maximizing energy transfer efficiency but also for suppressing energy losses and enhancing emission purity.^41–43^ Accordingly, accurate regulation of dopant concentration is identified as a critical factor in improving both the efficiency and long-term operational stability of OLED devices. Nevertheless, this optimization must be carefully balanced. While higher doping levels improve energy transfer, they can also exacerbate molecular aggregation, leading to aggregation-induced quenching and exciton loss.^44–47^ Therefore, an optimal trade-off must be struck to maximize efficiency while mitigating deleterious intermolecular interactions. Interestingly, despite variations in doping concentrations, all devices exhibit remarkably consistent EL spectra with minimal spectral shift, maintaining CIE coordinates around (0.35, 0.55). This spectral stability, along with the presence of a single dominant emission peak, indicates robust and uniform energy transfer from the BCCOX host to the 4CzIPN emitter. Such behavior strongly supports the conclusion that BCCOX serves as an energetically and structurally compatible host material for green TADF emitters, effectively maintaining color stability and emission integrity across a range of operating conditions.

**Fig. 6:**
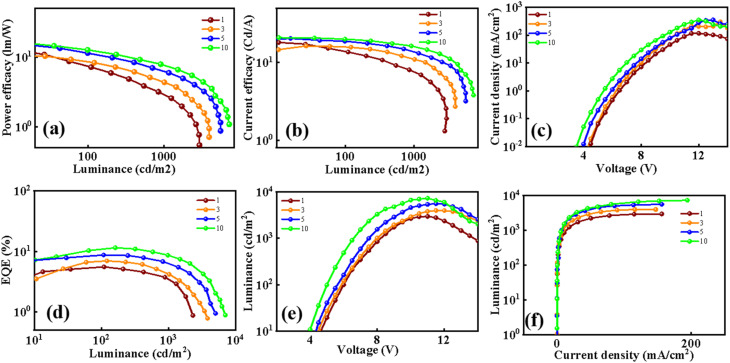
Electroluminescent characteristics of OLED devices employing 4CzIPN as the emissive dopant within the BCCOX host matrix are presented: (a and b) current efficacy power efficiency as a function of luminance for dopant concentrations of 1, 3, 5, and 10 wt%; (c) current density *versus* voltage; (d) EQE–luminance characteristics; (e) luminance as a function of voltage; and (f) luminance plotted against current density.

OLED devices fabricated with the newly developed BCCOX host exhibit excellent electroluminescent performance, especially when incorporating 10 wt% of the TADF emitter 4CzIPN. At this optimal doping level, charge carrier aggregation is effectively minimized, resulting in improved electron–hole balance and, consequently, more efficient radiative recombination within the emissive layer. As depicted in Fig. 6d, the device incorporating 10 wt% 4CzIPN exhibits superior EQE compared to devices with lower or higher dopant levels. The emission profile remains spectrally stable across varying applied voltages, exhibiting a negligible shift in peak wavelength and maintaining consistent chromaticity, which suggests excellent operational stability under electrical bias. Additionally, the voltage-dependent current density and luminance curves (Fig. 6c and f), along with the current efficiency (CE) and power efficiency (PE) as functions of luminance (Fig. 6a and b), further confirm the device's favorable performance characteristics. Notably, the EQE–luminance relationship indicates that the peak efficiency occurs at the 10 wt% doping level. A further increase in dopant concentration leads to performance degradation, likely due to enhanced non-radiative decay pathways and disruption of charge balance within the emissive layer. The optimized OLED configuration achieves a peak EQE of 10.4%, a CE of 20.2 cd A^−1^, and a PE of 12.7 lm W^−1^, while operating at a relatively low turn-on voltage of 3.9 V (as summarized in Table S1), resulting in high-quality green emission. These findings underscore the critical importance of precise dopant concentration control in host–guest systems. The 10 wt% 4CzIPN-doped BCCOX matrix is particularly effective in promoting efficient exciton formation and confinement, thereby enabling high-efficiency of low-voltage-driven light emission.

**Fig. 7:**
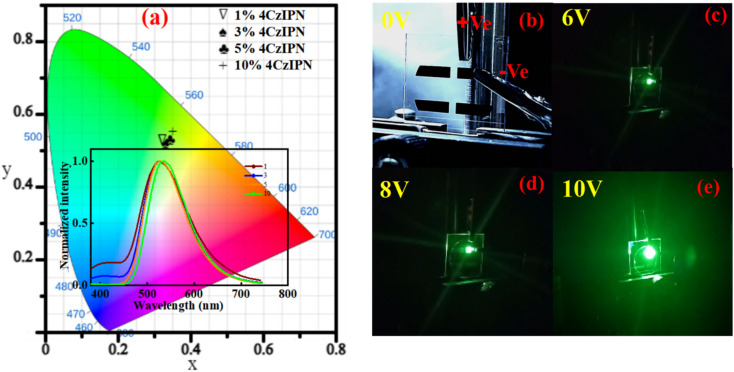
Electroluminescence properties of OLED devices based on 4CzIPN-doped BCCOX host systems: (a) CIE color coordinates illustrating the emission hues associated with varying external quantum efficiencies for devices using the BCCOX host, accompanied by inset electroluminescence spectra recorded at different 4CzIPN doping levels (1.0, 3.0, 5.0, and 10.0 wt%); (b) optical image of the OLED device in the unbiased state; and (c–e) luminance images captured under different voltages.

## 4. Conclusions

In summary, this work introduces a newly engineered carbazole–benzocarbazole-derived material that offers structural flexibility together with desirable electronic–optical properties coupled with wide bandgap characteristics, and excellent transparency in the visible region. The synthesized compound benefits from a simple, economical preparation strategy and demonstrates strong thermal as well as photophysical robustness. In particular, this derivative demonstrates remarkable thermal robustness, characterized by a decomposition temperature above 400 °C and a *T*_g_ near 194 °C. Moreover, it possesses a wide optical bandgap greater than 3.66 eV and rapid exciton decay dynamics, both of which are important parameters for enhanced efficiency in optoelectronic applications. Taking advantage of this favorable combination, the carbazole–benzocarbazole-based material was effectively employed as a host in green TADF OLEDs, at a concentration of 10 wt%, the resulting device exhibited efficient electroluminescent behavior, yielding a peak EQE of 10.4% and a maximum luminance of 7590 cd m^−2^.

## Author contributions

Sushanta Lenka: writing – original draft, methodology, investigation, conceptualization, visualization, formal analysis. Hsuan-Min Wang: device fabrication and characterization. Jayachandran Jayakumar: investigation, data curation. Daiva Tavgeniene: synthesis, investigation, Dovydas Blazevicius and Gintare Krucaite: investigation. Yu-Lin Chi: data curation, Saulius Grigalevicius: writing – review and editing, validation, supervision, funding acquisition, formal analysis. Jwo-Huei Jou: writing – review and editing, validation, supervision, resources, project administration, funding acquisition, formal analysis.

## Conflicts of interest

There are no conflicts of interest to declare.

## Supplementary Material

NA-008-D5NA01189B-s001

## Data Availability

All data supporting the findings of this study are available within the article and its supplementary information (SI). Additional datasets generated and analyzed during the current study are available from the corresponding author upon reasonable request. Supplementary information is available. See DOI: https://doi.org/10.1039/d5na01189b.
