# Supplementary material for: Carbazole–benzocarbazole fragments having derivative as very efficient host material for TADF based OLEDs
Source: Nanoscale Adv. 2026 Feb 16;8(6):1992–2002. doi: 10.1039/d5na01189b (PMC12917368; doi:10.1039/d5na01189b)
Supplement: NA-008-D5NA01189B-s001 [file NA-008-D5NA01189B-s001.pdf]

Supplementary Information (SI) for Nanoscale Advances.
This journal is © The Royal Society of Chemistry 2026

Carbazole-benzocarbazole fragments having derivative as very efficient host material for TADF based OLEDs

Sushanta Lenka[1], Daiva Tavgeniene[2], Hsuan-Min Wang[1], Jayachandran Jaykumar[1], Dovydas Blazevicius[2], Gintare Krucaite[2], Yu-Lin Chi[3], Saulius Grigalevicius[2],* and Jwo-Huei Jou [1],*

[1]Department of Materials Science and Engineering, National Tsing Hua University, No. 101, Section 2, Guangfu Rd., East District, Hsinchu, Taiwan, 30013

[2]Department of Polymer Chemistry and Technology, Kaunas University of Technology, Radvilenu Plentas 19, LT50254 Kaunas, Lithuania

[3]Department of Chemistry, National Tsing Hua University, No. 101, Section 2, Guangfu Rd., Hsinchu 30013, Taiwan

Corresponding Authors:

S. Grigalevicius, Email: saulius.grigalevicius@ktu.lt

J.-H. Jou, Email: jjou@mx.nthu.edu.tw

Table of content

1. Experimental section

1.1. Instruments

1.2. Materials

1.3. Spectra of BCCOX

2. **Characterizations**

3. Device fabrication

1. Experimental section

1.1. Instruments

Ultraviolet-visible (UV-vis) absorbance measurements were performed using an HP-8453 diode array spectrometer. This instrument covers an extended spectral range spanning from 250 to 800 nm, which encompasses both the near-ultraviolet and visible regions. To obtain precise transmittance measurements, the same spectrometer was utilized, capturing data across the entire visible spectrum, ranging from 380 to 780 nm. These measurements enabled detailed insights into the optical properties of the samples under study.

For ultraviolet photoelectron spectroscopy (UPS) analysis, the U-3010 spectrometer was employed with a specific focus on characterizing the electronic properties of host materials. This analysis was crucial for determining the work function and probing the highest occupied molecular orbital (HOMO) levels, thereby providing important information on the electronic structure.

Finally, atomic force microscopy (AFM) was carried out using the Bruker Dimension ICON instrument. This technique enabled the mapping of surface topography at the nanoscale, offering insights into surface roughness and other critical surface characteristics necessary for comprehensive material characterization.

1.2 Materials

All chemicals, which were of analytical grade, were directly used without any additional purification steps. Tris(4-carbazoyl-9-ylphenyl)amine (TCTA), 2,2',2"-(1,3,5-benzinetriyl)-tris(1-phenyl-1-H-benzimidazole (TPBi), lithium fluoride (LiF), tris(2-phenylpyridine)iridium (Ir(ppy)$_3$) were obtained from Shine Materials. Poly(3,4-ethylene dioxythiophene)-poly(stryenesulfonate)

(PEDOT: PSS), sodium hydroxide (NaOH), sodium titanate hydrated ($Na_2Ti_3O_7.mH_2O$) and hydrazine hydrate ($N_2H_4 \cdot H_2O$) were purchased from Sigma Aldrich and the filter paper was purchased from Merck Millipore.

9H-Carbazole (**1**), 11H-benzo[*a*]carbazole, *N,N*-dimethylformamide (DMF), ethylmethylketone, acetic acid (CH_3COOH), tetrahydrofurane (THF), 18-crown-6, copper (Cu), copper(I) iodide (CuI), K_2CO_3, KOH, KI, KIO_3, and Na_2SO_4 were purchased from Aldrich and used as received.

3-Iodo-9H-carbazole (**2**) was synthesized according to the procedure described in literature [1].

3,3-Di[3-iodocarbazol-9-ylmethyl]oxetane (**3**) was performed according to the same method, which we have described earlier [2].

3,3-Bis[3-(benzo[a]carbazol-11-yl)carbazol-9-ylmethyl]oxetane (BCCOX). Cu (0.12 g, 1.89 mmol) and K_2CO_3 (0.52 g, 3.77 mmol) were added to a solution of compound **3** (0.5 g, 0.75 mmol), 11H-benzo[*a*]carbazole (0.36 g, 1.66 mmol) and18-crown-6 (0.2 g, 0.76 mmol) in dry DMF (10 ml) under N_2 atmosphere. The mixture was stirred for 48 h at 155 ^oC. After cooling to room temperature, the reaction mixture was poured into water and extracted with chloroform. The combined extract was dried over anhydrous Na_2SO_4. The crude product was purified by silica gel column chromatography using the mixture of ethyl acetate and hexane (vol. ratio 1:10) as an eluent. Yield: 0.4 g of white product (64 %). MS (APCI+, 20 V): 847.18 ([M+H], 100%). ^1H NMR (400 MHz, $CDCl_3$, δ): 8.24 (s, 1H, Ar), 8.21-8.10 (m, 4H, Ar), 7.98 (d, 2H, *J* = 7.6 Hz, Ar), 7.87 (d, 2H, J = 8.0 Hz, Ar), 7.65 (d, 2H, J = 8.4 Hz, Ar), 7.54-7.42 (m, 6H, Ar), 7.39-7.19 (m, 12H, Ar), 7.12-7.06 (m, 2H, Ar), 6.99-6.86 (m, 2H, Ar), 4.87-4.69 (m, 8H, 2×CH_2; 2×OCH_2). ^{13}C NMR (100 MHz, $CDCl_3$, δ): 142.78; 142.27; 141.19; 136.03; 133.50; 132.26; 129.15; 127.18; 127.06; 124.96; 122.17; 122.14; 121.45; 121.23; 121.04; 120.58; 120.25; 119.53; 119.21; 110.54; 109.88; 109.13; 50.83; 47.77.

1.3 Spectra of BCCOX

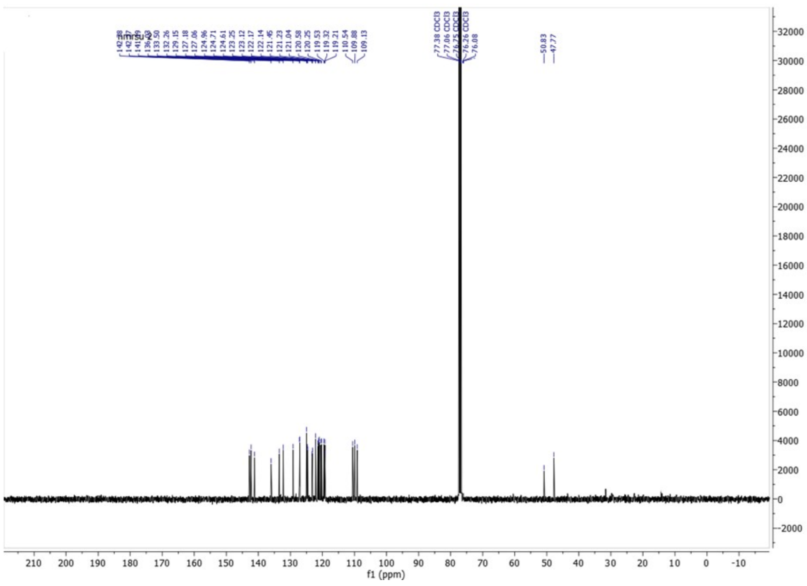

Figure S1. ^{1}H NMR spectrum of BCCOX

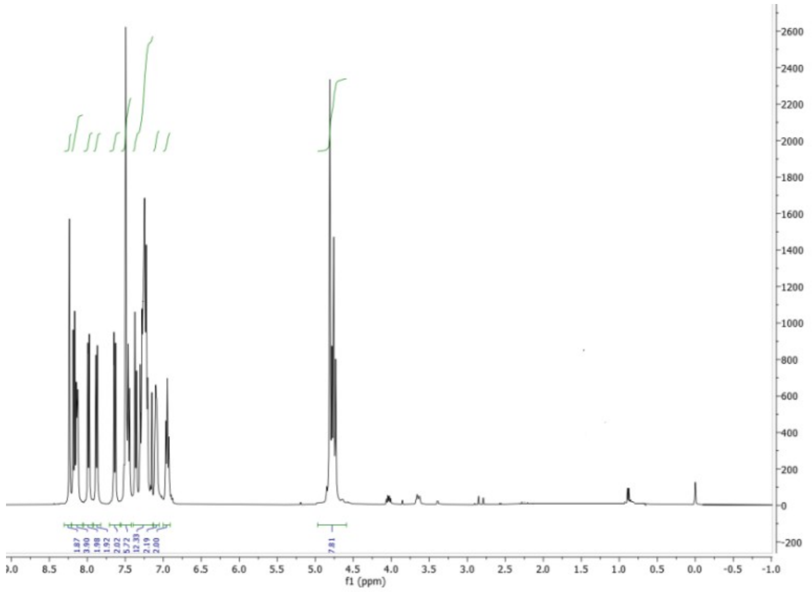

Figure S2.^{13}C NMR spectrum of BCCOX

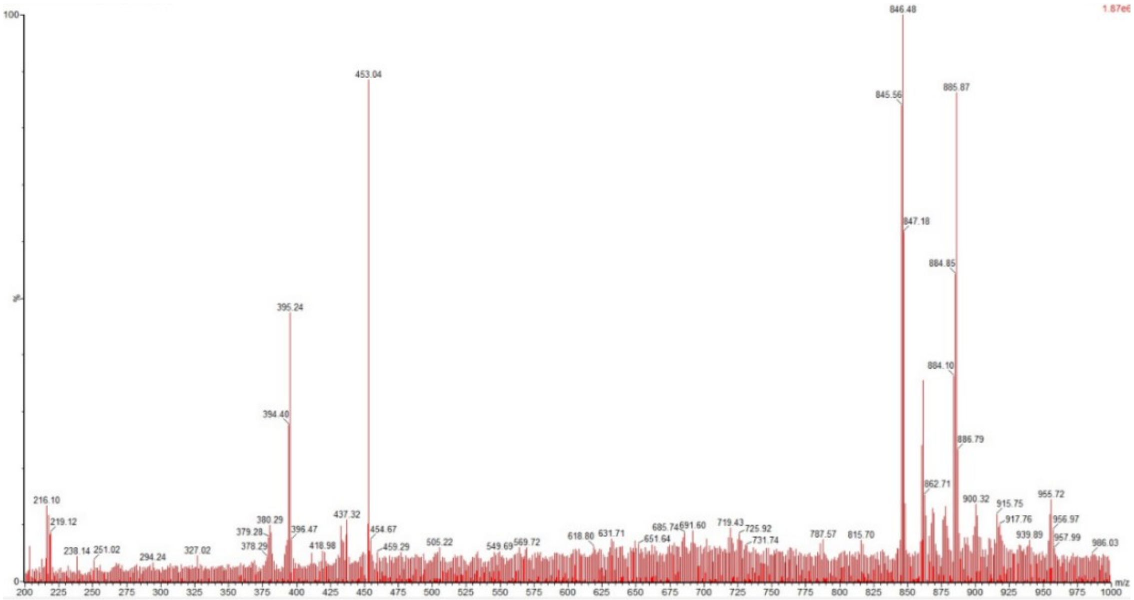

Figure S3. MS spectrum of BCCOX

2. **Characterization**

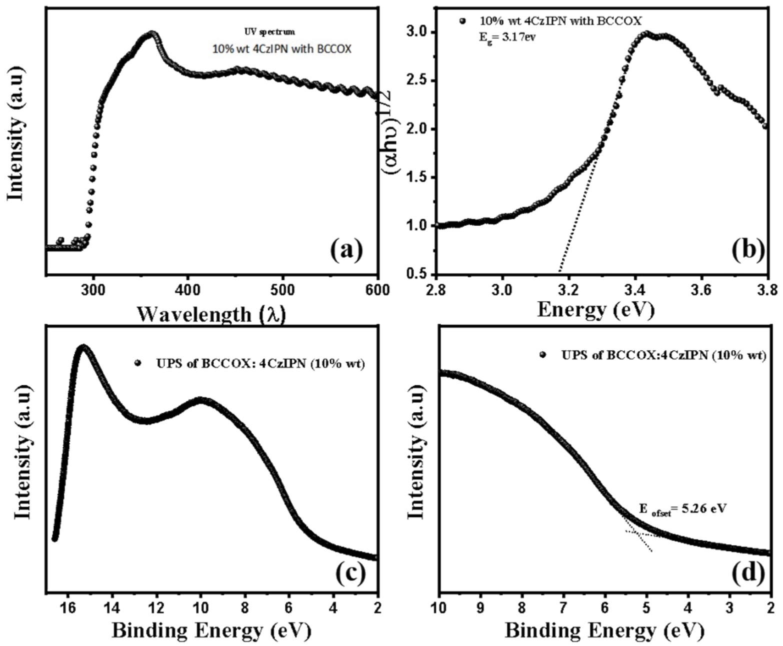

Figure S4. (a) and (b) UV analysis of composite film, (c) and (d) Work function and HOMO value of composite thin-film.

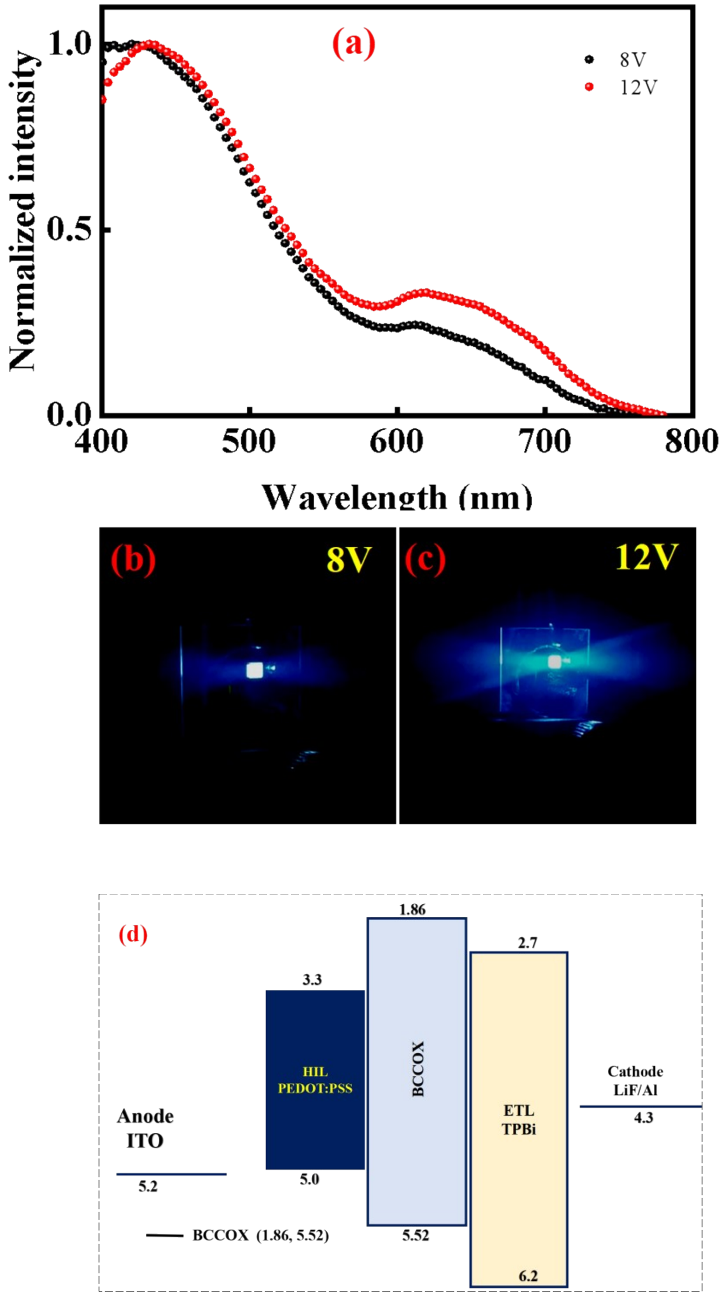

Figure S5. Electroluminescence characteristics of OLED devices of BCCOX host material only: (a) Electroluminescence spectra at two different voltages. (b) and (c) Devices operated at applied voltages of 8V and 12 V, respectively. (d) Schematic energy-level alignment of the individual functional layers for pure host materials OLED architecture

In this device architecture (see fig-S5d), we systematically investigated the electroluminescence (EL) characteristics of the host materials in their pristine form, without incorporating any dopant or emissive guest materials. The resulting EL spectra exhibited a broad emission profile, with peak emission positions comparable to those observed for commercially available host materials [3]. This behavior confirms that the intrinsic electroluminescence originates from the host materials themselves and is consistent with their expected electronic structure. In our studies, we have observed an evaluation of the electroluminescence properties of the pure materials, independent of any emitting dopants or host–guest interactions to assess and validate the inherent EL performance of the newly synthesized host materials under undoped conditions, providing a direct and unbiased comparison with established commercial hosts.

Table S1. A comprehensive list of the driving voltage, power efficacy, current efficacy, external quantum efficiency (EQE), CIE coordinates, and luminance of the OLED device based on BCCOX doped in 4CzIPN emitter.

**%wt of 4CzIPN emitter**	**Turn-on Voltage (V)**	**Operation Voltage (V)**	**Power Efficacy (lm/W)**	**Current Efficacy (cd/A)**	**EQE (%)**	**CIE**	**Max Luminance (cd/m^2)**
		**@ 100/1,000 cd/m^2**					
**1%**	**4.4**	**5.9/ 8.2**	**7.2/ 2.8**	**13.5/ 7.2**	**5.4/ 3.5**	**(0.32, 0.53)/(0.32, 0.51)**	**2,970**

**3%**	**4.1**	**5.6/ 7.5**	**9/ 4.5**	**16.1/ 10.8**	**6.9/ 4.3**	**(0.33, 0.52)/(0.33, 0.52)**	**4.380**
**5%**	**4.2**	**5.5/ 7.3**	**11.4/ 6.3**	**19.9/ 14.7**	**8.6/ 6.5**	**(0.34, 0.53)/(0.34, 0.53)**	**5.640**
**10%**	**3.9**	**4.9/ 6.5**	**13.2/ 7.5**	**20.6/ 15.5**	**10.4/ 7.2**	**(0.35, 0.55)/(0.34, 0.55)**	**7.590**

3. Device fabrication

A thorough, multi-step cleaning process was employed for pre-sputtered indium tin oxide (ITO) substrates with a sheet resistance of 25 Ω/sq. To begin, the substrates were treated in a soap solution to remove surface contaminants. They were then subjected to ultrasonic cleaning in two stages: first in acetone, followed by isopropyl butane, each for 40 minutes. The acetone stage was conducted at 50 °C, while the isopropyl butane stage was performed at 60 °C to ensure comprehensive removal of impurities. Following the ultrasonic cleaning steps, the substrates were placed in a pre-heated ultraviolet (UV) chamber for a 10-minute UV treatment to eliminate any residual moisture, creating an optimal surface condition for subsequent layer depositions. Concurrently, the hole-injection layer (HIL) and emissive layer (EML) were prepared. The emissive layer (EML) was fabricated by doping 4CzIPN at varying concentrations (1–10 wt%) into the BCCOX host matrix, with all components dissolved in a common solvent to ensure homogeneous mixing. The hole-injection and emissive layers were then spin-coated onto the

substrates at 4,000 rpm and 2,500 rpm, respectively, with each coating step lasting 20 seconds [4].

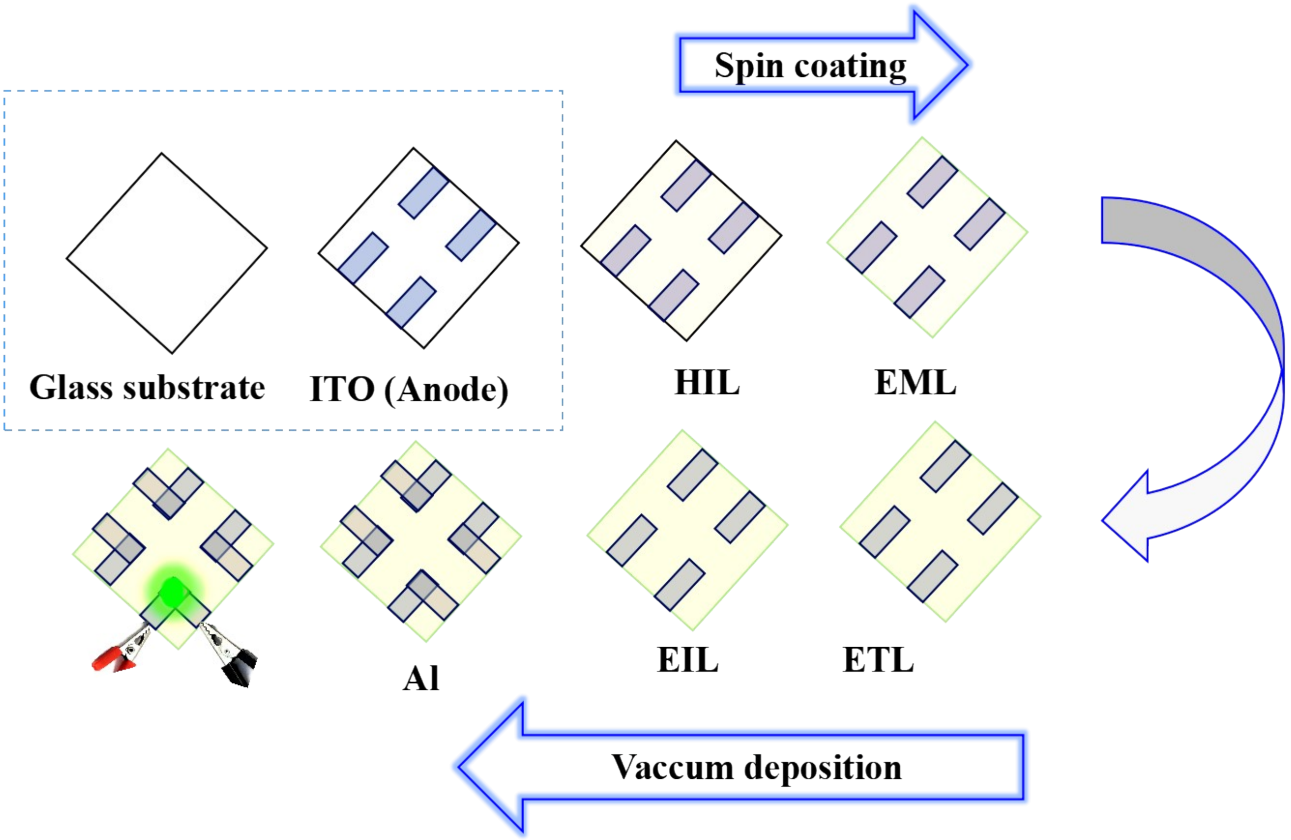

Figure S6. Fabrication process of solution process OLED

These processes took place in a nitrogen-filled glove box to prevent contamination from atmospheric oxygen or moisture. Once the spin-coating steps were completed, the substrates were transferred to a thermal evaporation chamber where the electron-transporting and electron-injection layers, along with an aluminum cathode, were deposited under a high vacuum of 10^{-6} torr. Throughout the evaporation process, a crystal sensor display was used to meticulously monitor the thicknesses of the layers and deposition rates, ensuring precision. Device performance was evaluated in an artificial dark room using a CS-100A system for current density-voltage-

luminance measurements, and a Photo Research SpectraScan PR-655 spectrophotometer was employed to measure current efficacy, luminance, and power efficacy characteristics. Additionally, current-voltage characteristics were recorded with a Keithley 2400 voltmeter, and all measurements were conducted on devices with an active area of 0.09 cm².

References:

[1] Tucker, S.H., 1926. LXXIV.—Iodination in the carbazole series. *Journal of the Chemical Society (Resumed)*, *129*, pp.546-553.

[2] S. Grigalevicius, L. Ma, G. Qian, Z. Xie, M. Forster, U. Scherf, *Macromol Chem Phys* 2007, *208*, 349.

[3] Behera, P.K., Lenka, S., Roy, M., Chen, F.R., Lin, W.Z., Jan, P.E., Lin, H.W., Jou, J.H. and Ammathnadu Sudhakar, A., 2024. Unveiling the yellow electroluminescence (EQEmax= 5.4%) of an unsymmetrical perylene diester imide. *ACS Applied Optical Materials*, *2*(11), pp.2221-2228.

[4] P. Gautam, S. Gupta, I. Siddiqui, W. Z. Lin, D. Sharma, A. Ranjan, N. H. Tai, M. Y. Lu, J. H. Jou, *Chemical Engineering Journal* **2024**, *495*, DOI: 10.1016/j.cej.2024.153220
